# Dolphins’ Willingness to Participate (*WtP*) in Positive Reinforcement Training as a Potential Welfare Indicator, Where *WtP* Predicts Early Changes in Health Status

**DOI:** 10.3389/fpsyg.2019.02112

**Published:** 2019-09-25

**Authors:** Isabella L. K. Clegg, Heiko G. Rödel, Birgitta Mercera, Sander van der Heul, Thomas Schrijvers, Piet de Laender, Robert Gojceta, Martina Zimmitti, Esther Verhoeven, Jasmijn Burger, Paulien E. Bunskoek, Fabienne Delfour

**Affiliations:** ^1^Animal Welfare Expertise, London, United Kingdom; ^2^Laboratoire d’Ethologie Expérimentale et Comparée EA 4443, Université Paris 13, Sorbonne Paris Cité, Villetaneuse, France; ^3^Parc Astérix, Plailly, France; ^4^Boudewijn Seapark, Sint-Michiels, Belgium; ^5^Attica Zoological Park, Athens, Greece; ^6^Department of Life Sciences and Systems Biology, University of Turin, Turin, Italy; ^7^Dolfinarium Harderwijk, Harderwijk, Netherlands

**Keywords:** animal welfare, bottlenose dolphins, positive reinforcement training, reward motivation, qualitative welfare measures

## Abstract

Welfare science has built its foundations on veterinary medicine and thus measures of health. Since bottlenose dolphins (*Tursiops truncatus*) tend to mask symptoms of poor health, management in captivity would benefit from advanced understanding on the links between health and behavioural parameters, and few studies exist on the topic. In this study, four representative behavioural and health measures were chosen: health status (as qualified by veterinarians), percentage of daily food eaten, occurrences of new rake marks (proxy measure of social activity), and willingness to participate (*WtP*) in Positive Reinforcement Training sessions as qualitatively measured by their caretakers. These data were collected multiple times a day, every day over the course of a year from a multi-facility, large sample size (*n_*dolphins*_* = 51), allowing powerful analyses of the relationships between measures. First, it was found that dolphins with a higher *WtP* score also had a significantly better health status, ate a higher percentage of their daily food, and a lower occurrence of new rake marks. In addition, the *WtP* score was significantly lower up to 3 days before the weekly veterinary diagnosis of a decrease in health state; the percentage of daily food eaten and new rake mark measures did not show any significant change before such a diagnosis. These results suggest that *WtP* in training sessions is a potential behavioural measure of dolphin welfare, and an indicator of early changes in the dolphins’ health state. We therefore suggest measurement of *WtP* as a more practical and non-invasive tool to support veterinary care and general management. More work needs to be conducted to elucidate the influence of social behaviour on health, and to identify other potential welfare indicators, but this long-term study has shown that qualitative measures can be both practical and valid when assessing dolphin welfare.

## Introduction

Welfare science, the objective measurement of animals’ affective states (Mason and Veasey, 2010; Hemsworth et al., 2015; Veasey, 2017), had its genesis in veterinary medicine (Dawkins, 2006). Health-related measures of individual welfare include assessing parameters such as body lesions, disease, immune response, body condition, and stress physiology. Over half a century ago when animal welfare issues started to capture the general public’s attention, health parameters were simple, accurate indicators of the severity of suffering of farm animals (Wemelsfelder and Mullan, 2014; Veasey, 2017). As reflected by the perception of ‘well-being’ in human society at the time, efforts were firmly focussed on measuring and improving the physical as opposed to psychological health of animals.

An increase in both general husbandry standards and our knowledge of human and animal affective states led welfare scientists to consider behavioural and later cognitive measures of animal welfare. The initial behavioural measures studied were those associated with poor health, primarily ‘sickness behaviour’ which usually manifests as lethargy, anhedonia, inappetence, and social isolation, and which can be a key indication of poor welfare in animals that have adapted to mask overt signs of injury and disease (Johnson, 2002; Millman, 2007; Sneddon et al., 2014). Other behavioural indicators include play, affiliative behaviour, aggression, abnormal and resting behaviours, and are now thought to be just as informative as health measures in terms of overall welfare (Dawkins, 2004; Joseph and Antrim, 2010). Welfare science has moved on from considering good welfare as simply the absence of suffering and is now focussed on defining and measuring positive affective states, with behavioural measures being a key element in their evaluation (Boissy et al., 2007; Yeates and Main, 2008). Support for “feelings-based” welfare definitions is strong, stipulating that health only impacts welfare if the animal’s feelings are affected (e.g., feeling sick), and therefore encouraging research on identifying welfare indicators associated with health conditions (Mason and Veasey, 2010; Watters, 2014; Hemsworth et al., 2015; Clegg et al., 2017b; Veasey, 2017).

This is not to say that health-related welfare measures are redundant: they are readily quantifiable, easy to standardise, and continue to be used in welfare assessment frameworks for a range of species (Welfare Quality^®^, 2009; Mononen et al., 2012; Clegg et al., 2015). Since there is no single, perfect measure of welfare, the most accurate method for scientists and managers who aim to measure the overall welfare of an animal or population is to develop a multidisciplinary assessment, comprised of a combination of health, behavioural and cognitive measures (Pritchard et al., 2005; Webster, 2005; Mason and Veasey, 2010). It is worth noting that these categories represent ‘animal-based’ measures, i.e., direct outputs that can be measured from the animal, and are thought to be more accurate welfare indicators than using ‘resource-based’ measures which focus on the resources we provide (Webster, 2005; Roe et al., 2011).

Among many different species, animal-based welfare measures developed so far have predominantly been quantitative (Wemelsfelder, 2007), where behavioural or health parameters are defined and measured among certain contexts. Some key individual welfare measures that have been validated against other indicators are: stereotypic behaviours (Mason and Rushen, 2006; Mason and Rushen, 2008), sustained agonistic behaviour (Shively et al., 1997; Papciak et al., 2013), close social bonds (Kikusui et al., 2006; Hennessy et al., 2009), cognitive bias (Mendl et al., 2009; Roelofs et al., 2016), skin condition (Pritchard et al., 2005; Mononen et al., 2012), stress response (Cockram, 2004; Palme, 2012) and body lesions (Broom, 1991; Robinson et al., 2018). There are many advantages to quantitative welfare measures: namely the high reliability and validity of the data (Martin and Bateson, 1986; Veasey, 2017). However, welfare scientists often aspire to measure inherently more holistic phenomena, such as attitude, personality and indeed when trying to integrate multiple indicators to evaluate overall welfare itself. Fortunately, human social science provides some guidance for how to most accurately measure these constructs, where qualitative measures have been used to capture this data for decades (Wemelsfelder, 2007). Recently, such qualitative techniques have been applied to animals in captivity and have been found to correlate to quantitative measures, indicating that they have “biological validity” (Rousing and Wemelsfelder, 2006; Wemelsfelder, 2007). Qualitative information also complements the quantitative through its interpretative role, providing comprehensive data on the situation which is critical when assessing welfare, but hard to realise (Wemelsfelder, 2007; Whitham and Wielebnowski, 2009). The most commonly used qualitative methodology thus far is the Qualitative Behavioural Assessment (QBA), where an observer evaluates an animal’s emotional expressivity by considering and integrating many aspects of its behaviour (Wemelsfelder, 2007). QBAs have been used to assess welfare during transport of farm animals (Stockman et al., 2011), in measuring their social behaviour (Rousing and Wemelsfelder, 2006), and were included in the well-received Welfare Quality^®^ assessments (Welfare Quality^®^, 2009), the largest Europe-wide project of its kind (Blokhuis, 2008). One of the key advantages of QBAs and general qualitative behavioural measurement is that data collection is feasible and inexpensive, and has therefore been shown to be preferred by animal managers for *in situ* monitoring of welfare (Napolitano et al., 2010; Maple and Perdue, 2013). However, despite these advances in qualitative measures of welfare, they are still often regarded as subjective and even anthropomorphic, and therefore are not as commonly used and thought to have lesser value than other presumed more objective measures (Hall et al., 2013; Wemelsfelder and Mullan, 2014).

Regular monitoring by animal caretakers themselves is thought to be the key to making actual improvements in welfare, especially in zoos and aquaria (hereafter zoos) (Maple, 2007). Zookeepers have a unique relationship with many of the animals under their care since they generally provide individualised care: they spend many hours each day in proximity to the animals, are their primary food providers, may engage in training with them, and sometimes have been present in their lives since birth (Hosey and Melfi, 2010; Szokalski et al., 2013). Therefore the keepers certainly have a high chance of capturing the subtle emotional and behavioural attitudes of the animals which might otherwise be inaccessible to researchers, and especially when using qualitative approaches (Weiss et al., 2006; Whitham and Wielebnowski, 2009; Gartner and Weiss, 2013). Inter-observer agreement on ratings between keepers in these studies has been shown to be high and the qualitative results have been significantly associated with quantitative data, as with farm animals (Whitham and Wielebnowski, 2009). Zookeepers’ potential to monitor and influence welfare is further strengthened by the fact that many zoos are increasingly engaging in Positive Reinforcement Training (PRT) with their animals in order to conduct husbandry procedures, cognitive enrichment and increase exercise. Conducting PRT increases the time spent with the animals, and has been shown to increase behavioural diversity, and to lower cortisol levels and stereotypic behaviours (Bloomsmith et al., 2003; Carlstead, 2009; Pomerantz and Terkel, 2009; Da Silva Vasconcellos et al., 2016).

Cetacean species kept in zoos around the world have recently been the focus of increased welfare discussions and research (Clegg et al., 2015; Brando et al., 2016; Butterworth, 2017; von Fersen et al., 2018), acting as proxies for the general debate on animals displayed in zoos. Regarding bottlenose dolphins (*Tursiops truncatus*), studies are starting to suggest single potential welfare indicators such as synchronous swimming (Clegg et al., 2017a), play (Serres and Delfour, 2017), and cortisol measurement (Ugaz et al., 2013; Monreal-Pawlowsky et al., 2017; Mercera, 2019). As with other socially complex animals such as primates (Morgan and Tromborg, 2007; Buchanan-Smith et al., 2013; Schino et al., 2016), close social bonds seem to promote positive welfare in dolphins, but on the same token social stress has strong potential to reduce welfare (Waples and Gales, 2002; Clegg et al., 2017a, b). Social tensions have even been reported as causing chronic health problems and death in a few cases, although data were anecdotal (Waples and Gales, 2002). Notably, when cetacean species are experiencing poor health, they often mask symptoms and ‘sickness behaviour’ until the pathology is well developed (Waples and Gales, 2002; Castellote and Fossa, 2006). There is therefore a need to identify any behavioural indicators which reliably signal the early stages of health problems (Clegg et al., 2017b). These might be related to the animal’s social behaviour, appetence or interaction with its environment. Thus far in the field of cetology, qualitative techniques have been used to measure dolphin personality, but not emotions or welfare. Such measures use the expertise of knowledgeable observers to integrate multimodal information over time and contexts in a way that one-dimensional quantitative data cannot (Meagher, 2009; Wemelsfelder and Mullan, 2014), are relatively cheap and easy to conduct, and are highly sensitive to the animals’ immediate environment (Fleming et al., 2013).

Captive cetaceans live in a unique environment regarding their relationship with their trainers/caretakers: they often spend hours daily completing tasks with familiar humans during training sessions, sometimes in close physical contact, within a type of working relationship framework (Brando et al., 2016; Clegg et al., 2018). The effect on dolphin behaviour of these training sessions, which may include shows, human-animal interactions, medical behaviours or research tasks, has been the focus of several welfare studies, with some concluding the animals view the training sessions positively (Trone et al., 2005; Miller et al., 2011; Sew and Todd, 2013) and others suggesting they have led to agonistic behaviours (Frohoff and Packard, 1995). Behaviour before predictable events such as training sessions, termed anticipatory behaviour, has been considered in other animals as well as dolphins: a recent study found that bottlenose dolphins positively anticipate both training sessions where food is provided, as well as familiar trainer interactions where no food rewards are given, with the authors suggesting that dolphins’ varying responses to both contexts could indicate their welfare state (Clegg et al., 2018). However, these studies are measuring the dolphins’ response to the sessions indirectly, through associated behavioural repertoires: there is no existing research measuring the animals’ level of motivation during the sessions. An animal’s motivation is defined as its tendency to engage in an activity and is adaptively shaped with the goal of increasing biological fitness, where the associated behaviours are often linked to positive and negative affective states (Manteuffel et al., 2009). Therefore motivation was considered a significant phenomenon to study in terms of welfare (Kirkden and Pajor, 2006). In modern facilities, Positive Reinforcement Training (PRT) sessions are exclusively used to condition the animals to tasks, within which they receive their daily rations of food (Kuczaj and Xitco, 2002; Brando, 2010). PRT sessions provide food rewards conditional on the performance of certain tasks, and thus a dolphin’s motivation during these sessions may relate to the acquisition of food or the performance of the tasks: previous studies on ‘contrafreeloading’ (preferring to work for rewards as opposed to obtaining them for free) suggest it is likely a combination of the two (Salamone et al., 1994; de Jonge et al., 2008). Although there is likely much inter- and intra-individual variation in the dolphins’ motivation for PRT sessions, several significant influencing factors can be suggested. For example, chronic stress and social isolation were found to decrease motivation for rewards in laboratory rats (*Rattus norvegicus*) (Kleen et al., 2006) and domestic pigs (*Sus scrofa*) (Pedersen et al., 2002) respectively. In other animals, health status has been shown to impact motivation to work for rewards, e.g., an increase in pro-inflammatory cytokines signalling an immune response, i.e., departure from good health, induces decreased motivation for rewards in various species (Larson, 2002; De La Garza, 2005). If the above findings were also applicable to captive dolphins, measuring the motivation in PRT sessions could be a valuable early identifier for health and chronic stress conditions, allowing proactive management and reducing the need for invasive interventions further down the line.

Despite increased efforts into measuring dolphin welfare, scientists have not yet applied multiple health and behavioural measures to a large sample size with repetitions over time: this is essential for capturing the true variation and validating welfare measures. While this is easily achievable in farm and laboratory studies (Blokhuis et al., 2013; Wemelsfelder and Mullan, 2014), in the zoo setting small population sizes and animal management logistics are often limiting factors (Botreau et al., 2009; Whitham and Wielebnowski, 2013). The only study that combined health and behavioural measures looked at 3 case studies, reporting in mainly anecdotes that the dolphins’ health problems were preceded by changes in social behaviour, appetence and their interactions with the trainers (Waples and Gales, 2002). If validated with a much larger number of animals, such measures would be extremely useful to managers for early prediction of dolphins in poor health and welfare [e.g., 48,49], and could also be applicable for wild animal research (e.g., understanding social context through rake mark prevalence, Cords and Mann, 2014). A multi-institutional study applying multidisciplinary welfare measures has recently been conducted with rhesus macaques (*Macaca mulatta*), successfully exploring the relationships between health and individual differences in personality, behaviour, and social status (Robinson et al., 2018). This study also used a concise set of representative welfare measures, which is an important step toward improving feasibility of assessments and increased industry uptake (Main et al., 2012; Wemelsfelder and Mullan, 2014).

The current study was therefore designed with the aim of collecting long-term, multidisciplinary welfare data from a large sample of captive bottlenose dolphins in multiple facilities, focussing on a small but representative number of qualitative measures that could be conducted by the caretakers themselves. The objectives of the project were to investigate four health and behavioural welfare measures, focussing on the potential links between motivation for rewards, health and welfare, and using both quantitative and qualitative approaches. The principal behavioural measure was willingness to participate during training sessions (“*WtP*”). This was chosen as the key potential welfare indicator based on findings that other animals’ motivation to work for rewards has been closely linked to their emotional, welfare and health state (Spruijt et al., 2001; De La Garza, 2005; Rygula et al., 2005), and given that the training sessions represented a significant element of the dolphins’ environment. We aimed to correlate *WtP* to three other measures in order to investigate the link between behavioural and health measures. Health status was qualitatively assessed as part of examinations by on-site marine mammal veterinarians, who gave a simple evaluation of the individual health status on a weekly basis. A qualitative score evaluating the social context was also developed, where the occurrence of new rake marks (caused by other dolphins scraping their teeth against one another) on the body was scored, since this has been previously been used as an indicator of aggression and social stress in dolphins (Waples and Gales, 2002; Scott et al., 2005; Marley et al., 2013). Lastly, a quantitative measure of the percentage of fish eaten out of the total offered was applied. This was included because a decrease in appetite, even if not severe anorexia, is a common indicator of poor animal welfare (Johnson, 2002; Millman, 2007; Sneddon et al., 2014), and has been correlated with social stress (Waples and Gales, 2002) and poor health (Johnson et al., 2009; Schmitt and Sur, 2012) in bottlenose dolphins specifically.

Our first hypothesis was that we would find correlations between some or all of the chosen four measures, supporting their use as indicators of welfare state, where poorer welfare would be reflected by lower willingness to participate in training sessions, poorer health status, higher occurrence of new rake marks, and a lower percentage of daily food eaten. Our second hypothesis was that *WtP* would predict the early changes in the Health score. This was supported by anecdotal evidence from a previous study where the dolphins participated less and less in training sessions shortly before health conditions were even diagnosed (Waples and Gales, 2002), and the fact that decreased motivation to work for rewards is correlated with decreased health and welfare in other mammal species (Larson, 2002; Pedersen et al., 2002; Rygula et al., 2005; De La Garza, 2005; Kleen et al., 2006), including humans (Yirmiya, 1997; Danna and Griffin, 1999; Fernet, 2013). We expected that the new rake mark occurrence and percentage of daily food eaten would correlate with the Health score due to the potential for links with social stress and sickness behaviour respectively. We did not expect that they would predict early changes in the Health score since in previous dolphin studies that looked at the link between these measures and the animal’s health, correlations have only been reported where health problems are severe (Dierauf and Gulland, 2001; Waples and Gales, 2002; Johnson et al., 2009).

## Materials and Methods

### Sample Population and Participating Facilities

Four dolphin facilities from four European countries participated in the project (Parc Astérix, France, *n*_dolphins_ = 7; Boudewijn Seapark, Belgium, *n*_dolphins_ = 8; Attica Park, Greece, *n*_dolphins_ = 8; and Dolfinarium Harderwijk, Netherlands, *n*_dolphins_ = 28), with a fifth facility aiding in the study’s early development stages (Planète Sauvage, France). This resulted in data being collected from a total of 51 bottlenose dolphins (25 males and 26 females, age range of 1–55 years, Table 1) over the year long study. The large sample size and long study period was necessary to capture a sufficient number of occurrences where the Health scores varied. At all four facilities, the dolphins were fed a variety of fish and squid species daily during multiple training sessions, with the total amount per day for each dolphin ranging between 1 (for the very young animals) and 12 kg. There were between 5 and 10 training sessions each day at all facilities, excluding the ‘free feed’ first and last sessions of the day where the dolphins were fed their full ration without any conditioned behaviours being asked. All participating facilities are accredited by the European Association for Aquatic Mammals (EAAM) and follow their standard guidelines (European Association of Aquatic Mammals, 2019), using exclusively Positive Reinforcement Training (PRT) where the dolphins received fish and/or secondary reinforcers (e.g., rubs, attention, toys) after performing conditioned behaviours, and where no punishment or negative outcome for their leaving the trainer’s presence (Laule et al., 2003). Training sessions could include training for public presentations, medical training, play sessions and research sessions, and on days when the facilities were open to the public there were between two and three public presentations. All these types of sessions and presentations were considered under the umbrella of ‘training session’ for our methods and analysis.

**TABLE 1:** Age and sex characteristics of bottlenose dolphin study population.

**Facility**	**Group**	***N*_total_**	***N* [juvenile:adult]**		**Age range [years]**		**Origin**
					
			**Females**	**Males**	**Females**	**Males**	**(*n*_wild caught:_*n*_captive born_)**
Parc Astérix	Parc Astérix	7	1:4	0:2	1−44	32–35	3:4
Boudewijn Seapark	Boudewijn	8	1:5	1:1	1–51	1–11	3:5
Attica Park	Attica	8	0:1	2:5	39–39	5–32	1:7
Dolfinarium Harderwijk	Dome	10	–	0:10	–	12–43	1:9
Dolfinarium Harderwijk	Delta 1	12	2:5	3:2	3–55	4–25	3:9
Dolfinarium Harderwijk	Delta 2	6	2:4	–	2–34	–	1:5
	TOTAL	51	6:19	6:20	1–51	1–43	12:39

*Juveniles: ≤10 years old; Adults: 11 years old or more. Ages presented are those taken approximately midway through the study (1st January 2017).*

The seven dolphins at Parc Astérix (Plailly, France) were housed in an outdoor pool conjoined to two indoor pools with a total volume of 3,790 m^3^ of water, where there was always free access to all pools. At Dolfinarium Harderwijk (Harderwijk, Netherlands), there were three groups of dolphins in two locations: the first location contained the ‘Dome’ group of 10 dolphins, kept in a network of seven artificial indoor and outdoor pools interconnected by gates and sluice channels, with a total water volume of 2,743 m^3^. The second location was called the “Delta” and was a set of natural seawater lagoons, with a total water volume of 11,380 m^3^. At the time of the study, the Delta contained two groups of dolphins, ‘Delta 1’ and ‘Delta 2.’ Delta 1 consisted of four interconnected pools (free access) with a total volume of 9,467 m^3^, and housed 12 dolphins. Delta 2 was made up of two connected pools with a total volume of 1,913 m^3^ and had six dolphins living there. At Boudewijn Seapark (Bruges, Belgium), the eight dolphins were housed in an indoor facility made up of a show pool and two smaller side pools, with in total volume of 2,050 m^3^ of water. In addition, a channel connected the front pool with the back pool, which had a total volume of 900 m^3^ and which was always available to the dolphins when training sessions were not taking place. At Attica Park (Athens, Greece), the outdoor pool system had a total volume of 4,600 m^3^ and consisted of four interconnected pools (1 large and 3 smaller holding pools) which were always open to the eight dolphins.

### Study Duration and Dataset

The study was carried out over a full year from September 2016 to October 2017. During this period, one dolphin was born (not included in analysis), and two dolphins of 55 and 32 years died (52 and 57 days of data were collected from these individuals before their death). Data was collected every day, multiple times daily, where the number of consecutive days of data taken for each dolphin varied between 272 and 365 days due to the study starting at different points at each facility (mean: 317 days; excluding two dolphins that died during study). This translates to a total of 15,635 days of data, with approximately 99,600 separate scores conducted on the dolphins’ *WtP* during training sessions.

### Behavioural and Health-Related Animal-Based Measures

The aim of the study was to collect multidisciplinary daily information to establish a large dataset of welfare-related data from a variety of dolphin facilities. The facilities were located in four different countries, where the animal care staff who would collect the information had a range of zero to moderate previous experience with scientific data collection. For this reason, the data collection methods had to be simple and able to be standardised across the international facilities. Therefore, qualitative scoring scales were used for three of the measures, with a fourth measure (Kg of fish eaten as a percentage of total offered) established as a quantitative measure since there was little room for error when recording these data (unlike attempting to quantify the animals’ behaviour).

#### Willingness to Participate, *WtP*

Animals’ motivation to work for rewards has been closely linked to their emotional, welfare and health state (Spruijt et al., 2001; De La Garza, 2005; Rygula et al., 2005), but had not yet been investigated in dolphins. Given that dolphins’ lives in captivity involve multiple daily sessions where the animals are conditioned to complete certain behaviours for rewards, we used this context to measure their motivation levels. We designed a measure to assess their Willingness to Participate (*WtP*) in sessions, with the aim of investigating whether it might be related to their general health and welfare. Qualitative scoring was the ideal method for measuring *WtP* during training sessions, where knowledgeable trainers could use a ‘whole-animal’ approach to assess the animal’s inclination to complete tasks for rewards, and record it easily multiple times per day. This is a similar approach to QBAs, which have been found to successfully measure the emotional state and welfare of terrestrial animals using qualitative approaches (Stockman et al., 2011; Rutherford et al., 2012; Fleming et al., 2013); however, there is a slight difference in that this study measured one aspect of the animal’s demeanour (i.e., its “willingness” or “eagerness”) as opposed to traditional QBAs which aim to assess the animal’s emotional expressivity as a whole (Wemelsfelder and Lawrence, 2001).

**FIGURE 1:**
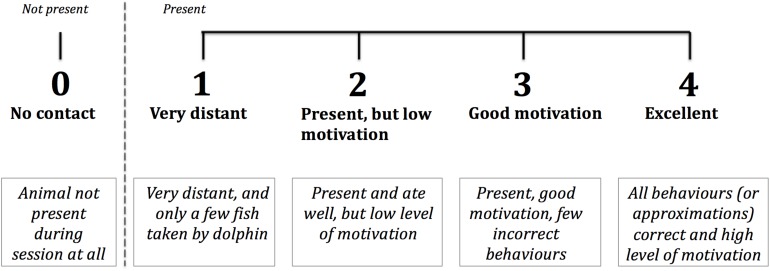
5-point Likert scale used by trainers in four facilities to score each dolphin’s Willingness to Participate (*WtP*) in multiple daily sessions over the year- long study.

The *WtP* score used in this study was a focal animal 5-point Likert scale, with the integers representing incremental grades of the dolphin’s motivation and enthusiasm during training sessions (Figure 1). The dolphin trainer who conducted the session with the animal assigned a score (or if multiple trainers, the person who spent the majority of the time with it), which had to be an integer and not a half score. Trainers were allowed to discuss their score choice with other caretakers, since the aim of the study was not to test individual trainer’s perceptions of the animals’ behaviour.

Before data collection started, an in-person meeting took place at each facility between the lead author (IC), the facility’s management, and the trainers who would be taking the scores. Reference videos were presented of each score on the scale, showing examples of indicative behaviours and accompanied by written explanations. Discussions followed on each of the scores, serving to consolidate the distinction between the scores and the aims of the study.

#### Health Score

**FIGURE 2:**
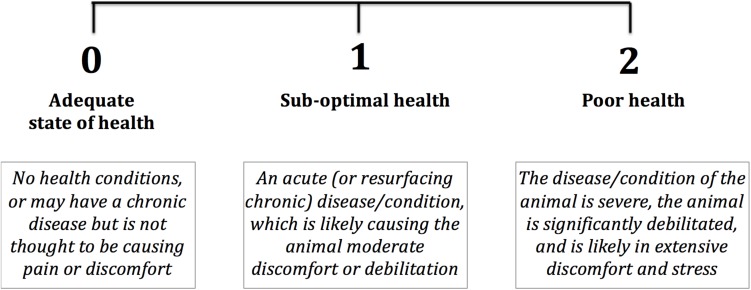
3-point Likert scale used by veterinarians in four facilities to score each dolphin’s health once each week over the year-long study.

The length of the study and many different participants involved meant that our aim was to standardise the measurement of the animals’ health as much as possible. Each facility had an associated veterinarian who performed an in-person health check on all animals each week, and so we sought to simplify and exploit this information for our study. Again, we developed a simple qualitative measure of health, a 3-point Likert scale (Figure 2), to maximise the likelihood that all veterinarians would score the animals’ condition in the same way. This approach is comparable to other multi-facility studies aiming to measure overall health in the long-term (Robinson et al., 2018). As with the *WtP* score, an in-person meeting between IC and each facility’s veterinarian took place before the study started, where the scale was discussed and the scores’ meaning agreed upon.

#### Percentage of Daily Food Eaten

In order to measure the dolphins’ appetite for food, a classic measure of health and welfare, we used a quantitative measure of the percentage of fish that each animal ate each day, out of the total offered (recorded in kg). The trainers weighed the amount of fish offered and eaten in each session, and totalled it for the whole day. “Offering” fish meant that over the course of the day the trainer was by the pool with the bucket of fish, giving the animal the opportunity to participate in the behaviours asked of it. An animal that scored <100% had chosen either not to approach the trainer for a proportion of the sessions that day, so that the fish could not be given, or had been present with the trainer and performed behaviours, but refused to eat all fish offered. All facilities conducted “free-feeds” in the morning and evening, and operated on the basis that if the animals chose not to perform behaviours in the training sessions, their full daily fish ration would be offered to them at the end of the day. Therefore, variance in the percentage of daily food eaten was primarily capturing those cases where the animal had refused the fish of its own volition. The total amount of fish offered per day to each dolphin varied with age, sex, facility, season, and on an individual basis, but was designed to maintain a healthy weight and optimum body condition.

#### Occurrence of Rake Marks

Measuring the presence of new rake marks on individual dolphins was used here as a proxy indicator for real-time changes in the social context of the group. During their daily sessions with the dolphins, the trainers visually scanned the animals’ bodies for any new rake marks that were visible. In order to standardise the evaluation of rake marks across facilities, we originally used a Likert scale with three categories: no new marks, a small number of new marks (about the size of one to two human hands), and a large number of new marks (covering the area of three hands or more). However, when the data were collected we had a very low sample size of the large rake mark category, which led us to treat the rake mark score as a binary “occurrence” in order to make it more meaningful. In another step to deal with the low variance in this score’s dataset, we transformed the daily rake mark score into a weekly score, where a score for any new marks on any day was treated as an occurrence and assigned a 1, and if not then a 0 was given.

### Ethics Statement

The study’s protocols were reviewed and approved by the welfare committees of the respective facilities; the data collected were solely observational and thus no specific permits were needed. The human participants’ involvement (as data collectors) did not require a permit as per the local legislation at each facility. All participating facilities were accredited and regularly inspected by the European Association for Aquatic Animal (EAAM), adhering to or exceeding their care and management standards (European Association of Aquatic Mammals, 2019).

### Statistical Analysis

Statistical analyses were done with the programme R, version 3.5.2 (R Development Core Team, 2018). Samples used for analyses were repeated measurements of different scores based on 51 bottlenose dolphins living in 6 different groups at 4 different facilities; see details in Table 1. For all dolphins, we calculated weekly averages of daily measures of *WtP* scores (ranging from 0 to 4, see Figure 1; on a numerical scale due to weekly averages; *total n*_weekly  values_ = 2,247) and of percentage of daily food eaten (% values; *total n*_weekly  values_ = 2,247) were averaged weekly. For analysis of the new rake mark score we used a categorisation of ‘0 = no new rake marks’ and ‘1 = new rake marks’ for each week (*n*_weekly  values_ = 2,247). Furthermore, a single Health score was available each week (*total n*_weekly  values_ = 2,238). In some rare cases, when the veterinarian visited the facility for several times per week, we used the weekly median value of the score, and thus the weekly averages remained on an ordinal scale.

**FIGURE 3:**
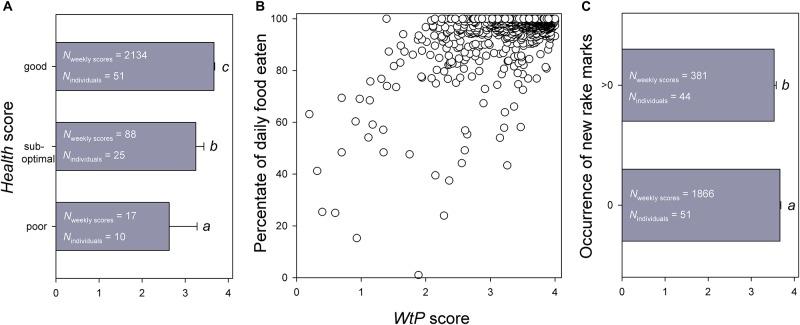
Associations of the average weekly *WtP* score (reflecting the dolphin’s willingness to participate in Positive Reinforcement Training) with **(A)** the Health score assigned by the veterinarian during weekly visits, **(B)** the percentage of daily food eaten, and **(C)** the occurrence of new rake marks on the dolphin. All associations were statistically significant, see text for details. Different letters beneath the vertical bars in **(A,C)** highlight significant differences between categories within each figure.

In a first step, we analysed associations between the *WtP* score (dependent variable) and either (a) the Health score, (b) the percentage of daily food eaten or (c) the occurrence of new rake marks (dependent variables in separate statistical models. For (a), due to the ordinal structure of the dependent variable, we used a cumulative link mixed-effects model for ordinal data using the *clmm* function provided by the R package *ordinal* (Christensen, 2015). *Post hoc* comparisons (after sequential Bonferroni correction, Holm, 1979), as shown in Figure 3A, were done using principally the same model, but based on a subset of the data restricting the analysis only to 2 of the ordinal categories of the dependent variable. For Figure 3B, we used a generalised linear mixed-effects model (GLMM) for proportional data, and for Figure 3C we used a GLMM for binomial data. This was done by using *glmer* function of the R package *lme4* (Bates et al., 2017). For all models, we included the identity of the dolphin as a random factor to account for the individual-based repeated measurements across consecutive weeks. We also included the identity of the facility and the identity of the group as further random factors to account for potential effects of the same origin (either facility origin or group origin) of the animals. Furthermore, we tested all remaining, possible associations between the different score variables using the different functions described above (see section Results for details).

**FIGURE 4:**
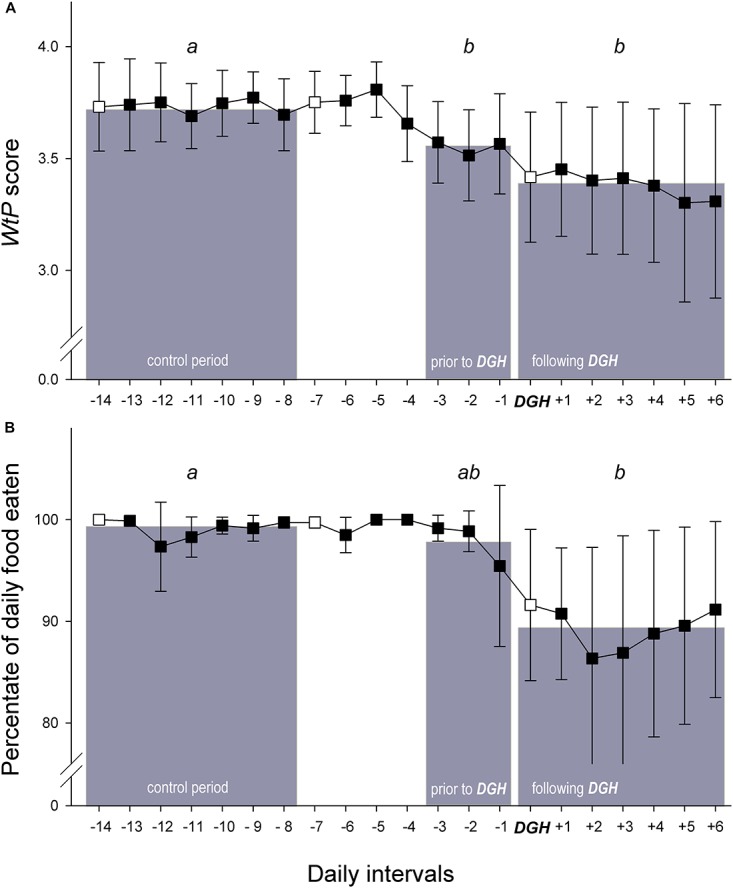
Changes across time in **(A)** the *WtP* score (reflecting the dolphin’s willingness to participate in Positive Reinforcement Training) and **(B)** the percentage of daily food eaten before and after a departure from good health (‘DGH’) in the dolphins (*N* = 26 individuals) was diagnosed during the weekly visit of the facilities’ veterinarians, defined as a decrease of the Health score from 0 to 1 (*N* = 22 individuals) or from 0 directly to 2 (*N* = 4 individuals). The days of the veterinarians’ weekly visits are indicated by an empty circle. Grey bars in background show the mean values of the different time periods, on which the statistical comparisons were based; see text for details. Statistically significant differences between the three periods are highlighted by different letters (Bonferroni-corrected comparisons *post hoc* to LMM with parametric bootstrapping, see text for details).

In a second step, we analysed whether the three different scores, *WtP*, percentage of daily food eaten and the occurrence of rake marks already showed any apparent changes shortly before the veterinarian determined a ‘departure from good health’ (DGH) in a dolphin. We considered DGH incidents as where the Health score given by the veterinarian decreased from 0 to 1, or 0 to 2, and no other such case preceded for at least 3 months previously (and therefore cases where scores decreased from 1 to 2 were not included). These criteria were established with the aim of analysing independent health issues, where the previous medical history of the animal was known (i.e., excluding cases where an animal was in decreased health at the start of the study period) and allowing us to set a control period for comparison. Based on these criteria, we included *n* = 26 DGHs from 26 different dolphins (juveniles < 10 years: 3 males, 4 females; adults: 12 males, 7 females) originating from five different groups at all four facilities into this analysis. From these 26 cases, there were 22 cases where the score decreased from 0 to 1 and four cases where the score went from 0 directly to 2. We considered restricting the analysis to only the 22 cases where the Health score decreased from a 0 to 1 to increase standardisation, but we principally found the same significant results, and so decided to keep in all cases of DGHs to maintain a larger sample size. Daily *WtP* scores and the percentage of daily food eaten were averaged over a 3-day period prior to and over a 7-day period following the diagnosis of a DGH by the veterinarian, to test whether these parameters could indicate the onset of DGHs. Furthermore, we assigned a 7-day control period ending 1 week prior to the diagnosis of the departure from good health (see grey bars in Figures 4A,B). For the occurrence of new rake marks, we assigned the absence/presence (binomial data structure) during the different periods. For the *WtP* score and the percentage of daily food eaten we ran a linear mixed-effects model LMM, and for the occurrence of new rake marks we ran a generalised linear mixed-effects model GLMM for binomial data, by comparing the three periods using *lmer* and the *glmer* functions of the R package *lme4*, respectively (Bates et al., 2017). In case of significant difference, we calculated pair-wise *post hoc* comparisons between the different periods (after sequential Bonferroni correction, Holm, 1979) using the same kind of model but restricted to subsets of the data. Models always included the identity of the dolphin and the identities of the facility and of the group as random factors. As the distributions of the *WtP* score and the percentage of daily food eaten were different from normal, we calculated *P*-values by parametric bootstrapping, a resampling technique which does not have any specific requirements about the distribution of the data. This was done using the R package *afex* (Halekoh and Højsgaard, 2014). For all models, we tested for potential effects of age class (juvenile vs. adult) and sex, and the interactions of these two factors with period (factor with 3 levels).

## Results

### Associations Between *WtP* and Health Scores, Percentage of Food Eaten, and Occurrences of New Rake Marks

Higher *WtP* were significantly and positively associated with the veterinary Health score (Cumulative mixed model for ordinal data: $\chi_{1}^{2}$ = 108.550, *β*_poor/sub–optimal_ = 5.215 ± 0.876 SE, *β*_sub–optimal/good_ = 7.780 ± 0.943 SE, *P* < 0.001; *post hoc* comparisons in Figure 3A) and with the percentage of daily food eaten (GLMM for proportional data: $\chi_{1}^{2}$ = 63.619, *β* = 1.094 ± 0.137 SE, *P* < 0.001; Figure 3B), and were significantly and negatively associated with the occurrence of new rake marks (GLMM for binomial data: $\chi_{1}^{2}$ = 13.527, *β* = −0.328 ± 0.089 SE, *P* < 0.001; Figure 3C). That is, animals with a higher *WtP* score were in a significantly better health status, took a significantly higher percentage of the food they were offered, and had a significantly lower probability of carrying new rake marks.

Furthermore, animals with a higher Health score also showed a significantly higher percentage of daily food eaten (Cumulative mixed model for ordinal data: $\chi_{1}^{2}$ = 127.080, *β*_poor/sub–optimal_ = 4.367 ± 0.469 SE, *β*_sub–optimal/good_ = 7.180 ± 0.591 SE, *P* < 0.001). However, there were no significant associations of the animals’ Health score (GLMM for binomial data: $\chi_{1}^{2}$ = 0.742, *β* = −0.070 ± 0.081 SE, *P* = 0.389) or the percentage of daily food eaten ($\chi_{1}^{2}$ = 1.728, *β* = −0.091 ± 0.069 SE, *P* = 0.189) with the occurrence of new rake marks.

### Changes in Different Measures Related to the Occurrence of a Diagnosis of a ‘Departure From Good Health,’ DGH

For analysis, we averaged the daily *WtP* scores, percentage of daily food eaten, and occurrence of new rake marks to be able to compare two periods: a 3-day period prior to, and a 7-day period following, the diagnosis of a ‘departure from good health’ (DGH) by the veterinarian. Furthermore, we assigned a 7-day control period ending 1 week prior to the diagnosis of the DGH (see grey bars in Figures 3A–C).

There were significant differences between the three periods assigned with respect to the *WtP* score (LMM with parametric bootstrapping: *P* = 0.004; Figure 4A). *Post hoc* comparisons (given in Figure 4A) revealed that *WtP* scores during the control period were significantly higher than during the period prior and during the period after the diagnosis of a departure from good health (‘DGH’ in Figure 4) by the veterinarian. In other words, a significant decrease in the *WtP* score had already occurred prior to the veterinarian’s visit during which decreased health was detected.

In addition, there were some significant differences between the periods with respect to the percentage of daily food eaten (LMM with parametric bootstrapping: *P* = 0.007; Figure 4B). As shown by *post hoc* comparisons in Figure 4A, the percentage of daily food eaten during the control period was significantly higher than during the period after the diagnosis of a DGH. However, the period prior to detection of decreased health did not differ significantly from the two other periods. That is, there is no significant support by the data that the percentage of daily food eaten was predictive of a DGH during the weekly visit of the veterinarian. Even a comparison between the percentage of food eaten on the last day before the diagnosis of a DGH (day -1) with the control period did not reveal any significant difference (LMM with parametric bootstrapping: *P* = 0.356).

The occurrence of new rake marks did not differ significantly during the control period and the periods prior to or following the detection of a DGH (GLMM for binomial data: $\chi_{1}^{2}$ = 1.033, *P* = 0.597).

There were no significant effects of sex and no significant effects of age class (juveniles versus adults) with respect to any of the four scores considered (all *P* > 0.10). Furthermore, there were no significant interactions between sex and period (all *P* > 0.10) or between age class and period (all *P* > 0.10), indicating that there were no sex-specific or age-class specific differences among the three periods considered with respect to any of the four scores.

## Discussion

The present study investigated how certain behavioural, health, social and food intake parameters might relate to overall dolphin welfare, through collecting long-term data from multiple dolphin groups and facilities. Firstly, it was found that animals with a higher Willingness to Participate (*WtP)* in training sessions had a significantly better health status, took a higher percentage of the food they were offered, and were less likely to have new rake marks. *WtP* therefore may be a good welfare indicator for captive bottlenose dolphins. In addition, we looked at the above welfare-related parameters around the time period where a departure from good health was recorded by veterinarians, and found that the *WtP* score had already significantly decreased prior to the veterinarian’s visit during which decreased health was detected, suggesting its use as an early predictor of health problems.

### Correlation of *WtP* With Health Scores, Percentage of Food Eaten, and Occurrence of New Rake Marks

The significant correlation of *WtP* data to the Health score, percentage of food eaten, and occurrence of new rake marks suggests that *WtP* does reflect some type of welfare-related state in dolphins: associations between several multidisciplinary parameters strengthens the power of welfare conclusions (Pritchard et al., 2005; Webster, 2005; Mason and Veasey, 2010). This approach is important because as pointed out in a recent study correlating multidisciplinary parameters of primate health and welfare, there are often complex interactions between individual animal characteristics and how they relate to welfare, which means there are often many alternative explanations for results (Robinson et al., 2018). A few past studies on dolphin welfare correlated two multidisciplinary parameters together to suggest a certain welfare state, but accepted that it remains difficult to conclude the causality or duration of the state (Ugaz et al., 2013; Clegg et al., 2017a). In order to further understand what *WtP* signified in this study, and what it didn’t, the correlative results of the other parameters were considered as well as the variation of *WtP* in relation to specific decreased health incidents.

None of the other parameters- Health scores, percentage of food eaten, and occurrence of new rake marks- correlated significantly with all other parameters like *WtP* did. This was surprising, since measures of appetite (i.e., percentage of food eaten) are often used as fundamental measures of welfare (Johnson, 2002; Millman, 2007; Sneddon et al., 2014). Nevertheless, dolphins with a higher Health score also ate a higher percentage of their food, which suggests that appetitive measures are specifically an important correlate of dolphin health, and which has been supported by evidence from previous studies (Johnson et al., 2009; Schmitt and Sur, 2012). The significant correlations of dolphins’ *WtP* with other welfare-related parameters suggests parallels to other species, where motivation to ‘work for rewards’ has been found to decrease with both poor health and welfare (Spruijt et al., 2001; Pedersen et al., 2002; De La Garza, 2005; Rygula et al., 2005). The fact that *WtP* was the only parameter to be correlated to all other measures suggests that it is closer to measuring overall welfare than other, more quantitative parameters such as percentage of food eaten, and its broad scope is more likely to capture a selection of the many animal-based indicators of welfare states. These advantages result from the use of qualitative methods for *WtP* measurement, where trainers rated each animal’s *WtP* each session, every day, on a 5-point Likert scale. Qualitative measurement of welfare and other holistic concepts such as animal emotionality are becoming increasingly favoured, in part due to the discovery that they are accurate and reliable but also because they have practical benefits (Wemelsfelder et al., 2000; Rutherford et al., 2012; Fleming et al., 2013). Qualitative measures such as those used in this study allows the harnessing of holistic knowledge from those caretakers who know the animals’ behaviour and welfare the best (Whitham and Wielebnowski, 2009; Phillips et al., 2017), and up until now has not yet been exploited in dolphin research, despite the many hours of daily close physical contact spent between animal and caretaker. Such a tool, which is simple to execute accurately, generates meaningful data and facilitates daily monitoring of the animals, would be very valuable to captive dolphin management (Clegg et al., 2015, 2017b).

A dolphin’s “Willingness to Participate” in training sessions could indeed be influenced by many variables, and it is likely that for some of the days and data points during our study, we might not have been measuring welfare but instead an individual variation in satiety, or perhaps a time when other events in the pool where far more rewarding than training sessions. However, this is where the importance of the sample size and study duration comes into play, in conjunction with the choice of parameters: firstly, the measures were chosen as they represent elements fundamental to any welfare state (i.e., health, social behaviour, appetite), which meant that explaining any trends should be more straightforward. For example, it is easy to comprehend that a dolphin who has poorer health, *and* is eating less of its food, *and* has more new rake marks is less willing to participate in training sessions because it is in a negative affective state. On the other hand, it would be counter-intuitive to conclude that the animals showing these same results were less willing to participate in training sessions because they were simply satiated. Secondly, the suggestion of *WtP* as a welfare indicator is supported by the large sample size (*n_dolphins_* = 51) and the sheer number of data points (almost 100,000 for the *WtP* score) which means that even if there are some false positives, any trends would be a result of the more logical explanations, corroborated by the caretakers’ expert opinions.

It is important to highlight here that the occurrence of new rake marks on its own may not signify poorer welfare states. Dolphins can receive rake marks in multiple types of ‘intense’ social activity: during agonistic interactions, but also during sexual behaviour and rough play (Scott et al., 2005; Marley et al., 2013). However, in line with discussions on this question in other studies (Scott et al., 2005; Marley et al., 2013), rake marks are much more likely to occur during behaviours involving aggression (which could also include coercive sexual behaviour, or play that turns aggressive) since more actual bites have been witnessed together with such activity (MacLeod, 1998; Parsons et al., 2003; Silva-Jr et al., 2005), and as a result, rake mark prevalence has been used in the literature as an indirect measure of aggression (Scott et al., 2005; Martin and Da Silva, 2006; Marley et al., 2013; Cords and Mann, 2014; Orbach et al., 2015). An original objective of this study was to measure the extent of new rake marks, which can reveal much about the associated social behaviour (MacLeod, 1998; Marley et al., 2013), but unfortunately we had a low occurrence of extensive new rake marks (score 2), and thus decided to analyse these data as simply a presence/absence measure. Although our results showed that *WtP* was significantly lower when there was an occurrence of new rake marks, the effect size was low (i.e., the difference of the mean values, see Figure 3C), and coupled with the aforementioned ambiguity regarding the link between rake marks and negative affect, we recommend that more work is conducted on this measure before it is used as a welfare indicator.

### *WtP* as an Early Indicator of Departure From Good Health (DGH)

Decreased health has long been used as a context for validating welfare parameters due to its relatively simple measurement and tangible implications (Dawkins, 1980; Broom, 1991; Fraser et al., 1997). Here, we took instances where the veterinarians has diagnosed the dolphins as showing a departure from good health (DGH, as defined in this study either a change in Health score from 0 to 1, or 0 to 2, where no other such case preceded for at least 3 months previously; Figure 2), and investigated how *WtP*, new rake mark occurrence and percentage of food eaten varied in the time prior to and following the DGH, and in comparison to a control period. We found that among these parameters, *WtP* was the only variable to significantly differ between the time prior to the DGH and the control period: it was significantly lower in the days prior to the DGH as compared to the control (where the animal was assumed to still be in good health, since one of our criteria for analysing DGHs was that the animals had not had a previous health issue for at least 3 months previously). This suggests that *WtP* can be used an early indicator of a DGH, since the animals started participated less in the sessions around the same time that the veterinarian made an official diagnosis of decreased health (we cannot conclude which one is more sensitive since *WtP* was measured daily, and the Health scores weekly). In addition, *WtP* was found to be significantly lower following DGH diagnosis than the period prior to it.

We also looked at how the other parameters varied in relation to the DGH: percentage of daily food eaten during the control period was significantly higher than in the period *after* DGH diagnosis, but levels just prior to the DGH did not differ significantly from the other two periods. Therefore, while the dolphins indeed ate significantly less in the week after the vet diagnosed them with a DGH, their appetite did not change significantly in the early stages of decreased health. This agrees with other studies showing that dolphins’ food consumption seems to decrease only when there is a serious health or social problem (Waples and Gales, 2002; Johnson et al., 2009; Schmitt and Sur, 2012). Our results suggest that the dolphins’ food consumption was not as sensitive to affective state change as *WtP*, which was already significantly decreased in the days prior to DGH diagnosis. Lastly, the occurrence of new rake marks did not differ between the control, prior-DGH and post-DGH periods, indicating that in the study population, new rake marks and therefore high arousal social interactions (e.g., aggression, sexual or rough play behaviour) were generally not a meaningful contributor or correlate to DGHs.

Poor health is notoriously difficult to diagnose in cetaceans since they are known to adaptively mask symptoms of pain and illness until the condition is severe and welfare is poor (Castellote and Fossa, 2006; Clegg and Delfour, 2018). However, small but significant changes in behaviour often occur as a health challenge establishes itself and animals enter what some call a ‘pre-pathological state’ (Moberg, 1985): it has been said that any measures of this subtle state may be the “most appropriate indicators of impaired well-being in that they identify (at an early stage) conditions that threaten tangible harm to the normal functioning of animals” [p197, 103]. Based on our results, we suggest *WtP* in training sessions as one of those indicators at least of decreased welfare due to impaired health, but possibly also for other negative affective states, e.g., linked to social issues.

A principal aim of our study was to gather a large amount of data in multiple facilities over a full year, to allow us to test enough repeats of different states. While the large sample size and long-term nature of the data allowed us to draw the conclusions above more confidently, the approach and especially the remote data collection element inevitably allowed for some risk of non-independence, which merits discussion. One source of non-independence may have been the fact that the veterinarians’ health diagnosis was influenced by the trainers telling them about the behaviour of the dolphins in the prior days, i.e., their *WtP*. This may have led to some DGHs diagnoses which would not have occurred if the veterinarian had not spoken to the trainers. This was unavoidable: in general this type of information-sharing is encouraged and necessary in dolphin facilities to ensure the best management of the animals. While this may have meant that strictly some of the prior decrease in *WtP* actually influenced the DGH diagnosis, it would not have changed the underlying reality of the situation which was that the veterinarian indeed believed a DGH was occurring and diagnosed it as such. That is, the non-independence may have increased the likelihood of DGH diagnoses but not increased false positive results, nor false negatives. The risk of non-independence in the other direction, i.e., veterinarians’ sharing views about the dolphins’ health state with the trainers which may have influenced their daily *WtP* scores, was likely to be very reduced since the veterinarians saw the dolphins much less regularly (once a week) than the trainers. Non-independence between health and the occurrence of rake marks would have been very unlikely since new marks would be considered a social group consideration to be managed by the training team and the veterinarian would not normally be told unless a period of sustained and excessive new marks occurred (personal communication). Similar to the *WtP* non-independence risk, trainers may have shared information about the percentage of food eaten with the veterinarian, but this would have only increased the likelihood of a correct DGH (or lack of) diagnosis, as opposed to increasing the chances of falsely diagnosing an animal as being in poor or good health.

### Significance for Dolphin Welfare Evaluation

Since cetacean species often mask symptoms of poor health until they are considerably compromised, it is all the more important to identify early predictors of any ‘pre-pathological states’ (Moberg, 1985; Fraser et al., 1997) that occur in order to ensure effective management and good welfare in captivity. From our results, *WtP* in training sessions significantly decreased in the 3 days prior to a DGH being diagnosed, suggesting that it could be used as an early indicator of decreased health, where in most facilities it may not be feasible for veterinarians to physically examine the animals every day. *WtP* was more sensitive indicator of decreased health than monitoring the animals’ daily food consumption, which is also often considered a failsafe measure of welfare (Johnson, 2002; Millman, 2007; Sneddon et al., 2014). The *WtP* measure designed in this study was simple, practical and non-invasive for the dolphins and trainers: if such scores (or similar) are taken already, we suggest their integration into the daily management routine. However, it is worth highlighting that to exploit the *WtP* scoring method and data fully, it is almost essential to take formal records of the scores and review the data regularly, i.e., at least calculating daily averages. The significantly different *WtP* scores in the pre-DGH, post-DGH and control periods only varied by an average of 0.2 (Figure 4A), which is not likely to be perceptible by a trainer, veterinarian or manager who simply glances over a set of scores (which had to be integers in this study) recorded each day. In addition, the approach and scores used here could be adapted for other animals in similar contexts of regular reward-based interactions, such a working dogs or riding horses.

Our results also showed that animals with higher *WtP* in sessions had significantly better health, took a higher percentage of the food they were offered, and had fewer new rake marks. *WtP* was the only parameter from the set of four used in this study to correlate to all the others, which suggests it is measuring an overall state that manifests through several multidisciplinary indicators. It is likely that dolphins’ *WtP* in training sessions is impacted by their health, appetite as well as their social environment, and these factors probably also interact in many ways both inter- and intra-individually: while this study does not determine the cause of the changing welfare states, the variance in dolphins’ *WtP* seems to effectively encompass these different welfare-related elements. Nevertheless, more research is certainly needed into why their *WtP* varies in relation to these factors, so that thresholds can start to be established and used to enhance management. While it has previously been advised to correlate several potential welfare measures to increase validity (Pritchard et al., 2005; Boissy et al., 2007), the complex inter-correlation of welfare-related variables means that future studies should even aim to use more than four measures to fully investigate the variance associated with health and welfare states (Robinson et al., 2018).

## Conclusion

This year-long, multi-facility study investigated the inter-correlations between four parameters related to dolphins’ behaviour, health and appetite which were designed to collectively measure their welfare. Firstly, we found that qualitatively measuring their Willingness to Participate (*WtP*) in training sessions seemed to reflect overall welfare state since it was the only one to vary with all other welfare-related measures, and was therefore a potential welfare indicator for captive dolphins. Further investigations in relation to incidents where the veterinarians’ had diagnosed a “departure from good health” (DGH) revealed that *WtP* in training sessions significantly decreased in the days prior to the veterinarians’ DGH diagnosis, suggesting it reflects early decreases in health. Furthermore, *WtP* was a more sensitive indicator of this ‘pre-pathological state’ than the change in percentage of food eaten. *WtP* and the other qualitative measures used in this study were shown to be not only meaningful and non-invasive, but also feasible for the animal caretakers to conduct on a daily basis. These results collectively suggest that measuring *WtP* by knowledgeable professionals in training sessions represents an accurate and comprehensive measure of dolphin welfare and may be useful to these animals’ management in captivity, although further work is still needed into the causal relationship between the contributing factors.

## Data Availability

The datasets generated for this study will not be made publicly available. The data is owned by the owners of the animals, but would be available with their permission.

## Ethics Statement

Ethical review and approval was not required for the animal study because only observational data was taken, with no changes whatsoever to the animals’ environment or management. Written informed consent was obtained from the owners for the participation of their animals in this study.

## Author Contributions

IC, HR, and FD contributed to the conception and structure of the manuscript. BM, SH, TS, PL, RG, MZ, EV, JB, and PB carried out and oversaw the data collection. IC and HR carried out the data analysis. IC wrote the manuscript. HR and FD edited the manuscript.

## Conflict of Interest Statement

The authors declare that the research was conducted in the absence of any commercial or financial relationships that could be construed as a potential conflict of interest.
